# Acetylcholine, Another Factor in Breast Cancer

**DOI:** 10.3390/biology12111418

**Published:** 2023-11-11

**Authors:** Juan P. Muñoz, Gloria M. Calaf

**Affiliations:** 1 Laboratorio de Bioquímica, Departamento de Química, Facultad de Ciencias, Universidad de Tarapacá, Arica 1000007, Chile; jpmunozb@academicos.uta.cl; 2 Instituto de Alta Investigación, Universidad de Tarapacá, Arica 1000000, Chile

**Keywords:** acetylcholine, estrogens, estrogen receptor alpha, muscarinic receptor, breast cancer

## Abstract

**Simple Summary:**

Our previous work established that organophosphorus pesticides increased acetylcholine (ACh) levels and promoted mammary gland tumor development in rats. This suggests that ACh could modulate ERα activity. In this study, we demonstrated that ACh has functional effects in breast cancer cell lines—specifically, activating the MAPK/ERK and PI3K/Akt pathways, inducing p-ERα, and eliciting its nuclear translocation. However, ACh did not induce the upregulation of estrogen-responsive genes, suggesting a mechanistic distinction from the effects of 17β-estradiol. Furthermore, ACh enhanced cell viability and induced the overexpression of specific EMT markers. These findings suggest that ACh and muscarinic receptors could be emerging regulators of breast cancer.

**Abstract:**

Acetylcholine (ACh) is a neurotransmitter that regulates multiple functions in the nervous system, and emerging evidence indicates that it could play a role in cancer progression. However, this function is controversial. Previously, we showed that organophosphorus pesticides decreased the levels of the enzyme acetylcholinesterase in vivo, increasing ACh serum levels and the formation of tumors in the mammary glands of rats. Furthermore, we showed that ACh exposure in breast cancer cell lines induced overexpression of estrogen receptor alpha (ERα), a key protein described as the master regulator in breast cancer. Therefore, here, we hypothesize that ACh alters the ERα activity through a ligand-independent mechanism. The results here reveal that the physiological concentration of ACh leads to the release of Ca^+2^ and the activity of MAPK/ERK and PI3K/Akt pathways. These changes are associated with an induction of p-ERα and its recruitment to the nucleus. However, ACh fails to induce overexpression of estrogen-responsive genes, suggesting a different activation mechanism than that of 17ß-estradiol. Finally, ACh promotes the viability of breast cancer cell lines in an ERα-dependent manner and induces the overexpression of some EMT markers. In summary, our results show that ACh promotes breast cancer cell proliferation and ERα activity, possibly in a ligand-independent manner, suggesting its putative role in breast cancer progression.

## 1. Introduction

Breast cancer is an unsolved public health problem and the most prevalent cancer type, with about 2.3 million new cases worldwide [1]. Although therapeutic methods and diagnostic tools have improved substantially in recent decades, breast cancer remains the leading cause of cancer-related mortality among women [2].

Breast cancer is a complex disease with several subtypes. The presence or absence of the estrogen receptor (ER), progesterone receptor (PR), and human epidermal growth factor receptor 2 (HER2) is typically used to categorize these subtypes [3]. Four more categories have been found by transcriptome-profiling study in addition to this classification system: basal-like, HER2-enriched, luminal A, and luminal B [4]. Notably, ER is overexpressed in approximately 80% of breast cancer cases [5]. This overexpression can make hormonal regulation a critical factor in tumor progression. As a result, antiestrogens and ER inhibitors are the most common treatments for ER-positive breast cancers [6].

ERs are a type of ligand-inducible transcription factor that belongs to the superfamily of nuclear hormone receptors and they regulate a plethora of physiologic processes in estrogen-target tissues, such as the breast, the uterus, and bone [7]. Two ER isoforms exist in vertebrates, ER alpha (ERα) and ER beta (ERß), encoded by two independent genes, *ESR1* and *ESR2*, respectively [8]. ERα can be activated by 17ß-estradiol (E2) through its direct binding or in the absence of a ligand, in a mechanism known as ligand-independent activation. This activation is triggered by the action of a plethora of regulatory kinases, in response to external stimuli such as growth factors, hormones, cytokines, and the neurotransmitter dopamine [9].

The mammary gland is not only exposed to hormones, but also to environmental agents that can modify its differentiation program [10]. Previously, we demonstrated that exposure to organophosphorus pesticides (OPs), parathion and malathion, led to mammary gland tumor formation in rats [11,12,13,14]. Since these pesticides are well-known cholinesterase inhibitors, a decrease in the activity of acetylcholinesterase, the enzyme that catalyzes the breakdown of the neurotransmitter acetylcholine (ACh), was also observed. The increases in serum ACh concentrations were concomitant with the formation of tumors in the mammary glands and an increase in muscarinic receptors [11]. Therefore, a hypothetical relationship between ACh and tumor formation is proposed.

ACh is a neurotransmitter in the central and peripheral nervous systems that plays a key role in learning, memory, autonomic control, and muscle contraction. However, it is not exclusive to the nervous system, since it was also identified in non-neuronal cells a long time ago [15,16]. ACh was demonstrated to be widely synthesized by airway epithelial cells, vascular endothelium, keratinocytes, glia, placental trophoblasts, and ovarian follicular cells. Some of these cell types have been reported to overexpress a cholinergic autocrine loop in which ACh stimulates cell growth [17]. Therefore, ACh was suggested to act not only as a neurotransmitter, but also as a growth factor [18]. In cancer cells, ACh is synthesized and plays a role in tumor development, promoting cell proliferation, invasion, vascularization, and migration [19,20,21,22].

ACh functions in the peripheral organs, activating two types of receptors—the ionotropic nicotinic (nAChRs) and the muscarinic (mAChRs) receptors [23,24]. mAChRs are metabotropic receptors belonging to the seven transmembrane superfamilies of receptors, whose group comprises five isoforms, termed M1–M5, encoded by five distinct genes (*CHRM1*-*CHRM5*). The signaling triggered upon ACh binding to mAChRs leads to a typical activation of Gq/11 proteins increasing the intracellular second messengers, diacylglycerol (DAG) and 1,4,5-trisphosphate (IP3). DAG activates protein kinase C (PKC), while IP3 triggers the release of intracellular Ca^+2^ into the cytosol [25]. The cytosolic increase of Ca^+2^ affects a wide variety of signaling pathways, such as the activation of PKC, Raf kinase, and MAPK ERK1/2 [23].

Several studies have demonstrated the expression of mAChRs in different tumor cell lines [26,27,28,29,30]. Particularly, the M3 receptor subtype has shown increased expression in human colon cancer when compared to normal tissue. The activation of this receptor has been shown to affect various aspects of cancer development, including cell proliferation, progression, and invasion [31,32]. In breast cancer cells, several studies have determined the overexpression of mAChR subtypes [33]. In this context, it has been demonstrated that stimulation of mAChRs in ERα-dependent breast cancer cell lines stimulates cell proliferation, protein synthesis, and angiogenesis through the MAPK/ERK signaling pathway [29,34,35]. Conversely, the treatment with atropine, a competitive antagonist of the M3 receptor, suppresses the epithelial–mesenchymal transition (EMT) [36]. Therefore, these lines of evidence have shown that mAChRs are involved in the progression of different tumors, and they have positioned themselves as attractive targets for therapeutic alternatives, especially for breast cancer [37,38,39].

In recent decades, some researchers have evaluated the effects of steroid hormones on mAChR gene expression. These studies have demonstrated that E2 can affect the mAChR gene expression in the central nervous system [40,41,42]. E2 was also shown to increase the mAChR expression in rabbit uterus and improve the response to carbachol, an M3 receptor agonist [43,44]. Therefore, these data suggest a link between the mAChR-triggered signaling and the activity of ERs in hormone-dependent tissues.

Therefore, given our preliminary results and the data provided by the literature, this work aimed to evaluate the role of ACh in enhancing the tumorigenic properties of breast cancer cell lines through ERα activation.

## 2. Materials and Methods

### 2.1. Chemical Reagents

Acetylcholine chloride (purity of ≥99%, TLC, Sigma-Aldrich, St. Louis, MO, USA). First, ACh was dissolved in water, filtered, and then aliquoted to create a stock solution 1 × 10^−3^ M. The solution was then stored in the dark at 4 °C. Analytical grade DMSO and ethanol were used to dissolve stock solutions of 1 µM of atropine (purity of ≥99%, TLC) and 10 µM of E2 (Sigma-Aldrich, St. Louis, MO, USA), respectively. The epidermal growth factor (hEGF) (provided by Thermo Fischer Scientific, Waltham, MA, USA) was reconstituted in PBS (stock, 1 mg/mL). Fulvestrant (Ful), (Abcam) was dissolved in DMSO (stock solution of 100 mM).

### 2.2. Cell Culture

Human breast cell lines MCF-10A (#C0006015), MCF7 (#C0006008), T47D (#C0006001), and MDA-MB231 (#C0006002) (Addexbio, San Diego, CA, USA). Dulbecco’s Modified Eagle’s Medium (DMEM) (Gibco, Carlsbad, CA, USA) were used to culture the MCF7 cell line; the medium contained 10 µg/mL of insulin (sc-360248, Santa Cruz Biotechnology Inc., Santa Cruz, CA, USA) and 10% (*v*/*v*) fetal bovine serum (Hyclone, Fremont, CA, USA). The T47D cell line was supplemented with 10% FBS, and it was cultured with the Roswell Park Memorial Institute (RPMI)-1640 (Hyclone, Logan, UT, USA). The MDA-MB231 cell line was maintained in DMEM high glucose (4500 mg/L glucose) from Gibco in Carlsbad, CA, USA, with 10% (*v*/*v*) FBS from Hyclone, Fremont, CA, USA). The cells were grown in hormone-reduced medium, which contained phenol red-free DMEM (Gibco, Carlsbad, CA, USA) supplemented with 5% charcoal-treated FBS (Gibco, Carlsbad, CA, USA), 1% sodium pyruvate, and 1% L-glutamine, for 48 h before and during the experiments to eliminate steroid stimulation. DMEM/F12 media (Gibco, Thermo Fischer Scientific, Waltham, MA, USA), 5% horse serum (Invitrogen, Carlsbad, CA, USA), 0.5 µg/mL EGF (Sigma-Aldrich, St. Louis, MO, USA), 0.5 µg/mL hydrocortisone (Sigma-Aldrich, St. Louis, MO, USA), and 10 µg/mL insulin (sc-360248, Santa Cruz Biotechnology Inc., Santa Cruz, CA, USA) were added to the MCF-10A cell line during growth. All cell media contained a mixture of antibiotics from Gibco, Carlsbad, CA, USA, consisting of 100 units/mL of penicillin and 100 g/mL of streptomycin. The cells were grown in flasks of 75 cm^2^ (Corning, Tewksbury, MA, USA) in a humid environment at a temperature of 37 °C with 5% CO_2_ saturation. When the cell adhesion area filled 80% of the culture dish, the media were replaced every two to three days, and the cells were passed enzymatically using trypsin and (0.05%) EDTA for five minutes at 37 °C.

### 2.3. Quantitative Real-Time PCR (q-RT-PCR)

Total RNA was isolated from cell cultures using TRIzol Reagent (Invitrogen, Waltham, MA, USA), according to a previous study [45]. The Qubit RNA BR test kit was used to quantify RNA samples, using the Qubit 4 fluorometer from Thermo Fisher, Waltham, MA, USA. Using the AffinityScript QPCR cDNA Synthesis Kit (Agilent Technologies, Inc., Santa Clara, CA, USA) and following the advised procedure, cDNA was produced, using 1 µg of total RNA. In a total volume of 20 µL, the reaction mix containing the first strand was created using 10 µL of 2× master mix, 0.1 µg/µL of Oligo dT, and 1.0 µL of AffinityScript RT/RNase Block enzyme mix. After that, the mixture was incubated for 30 min at 37 °C. Specific primers and the CFX96 real-time system (Bio-Rad Laboratories, Inc., Hercules, CA, USA) were used for real-time PCR quantification (Table 1). The Brilliant II SYBR Green q-RT-PCR 1-Step Master Mix (Agilent Technologies, Inc., Santa Clara, CA, USA) was used to create the qPCR mixture, which had a total volume of 25 µL. It contained 12.5 µL of 2X SYBR Green Master mix, 0.5 µL of each primer (400 nM), 10.5 µL of nuclease-free water, and 1 µL of template cDNA. For 40 cycles of qPCR, the thermocycling settings were 94 °C for 30 s, 58 °C for 20 s, and 72 °C for 20 s. The q-RT-PCR experiment was carried out in triplicate for each circumstance. To normalize expression levels, ß-Actin mRNA expression levels were employed. When expressing induction or inhibition as a fold change in comparison to normalized control values, the 2^−ΔΔCt^ method was used to provide the fold change [46]. Analyses of melting curves were carried out to verify proper amplification.

### 2.4. MTT Assay

The MTT assay is a widely used method for assessing cell viability and cytotoxicity, as previously described [45]. After dissolving in water and filtering through a 0.22 µm filter, MTT (3-4,5-Dimethylthiazol-2-yl)-2,5-diphenyl tetrazolium bromide was diluted to 1 mg/mL in a hormone-reduced medium before being used. In the experiments, the cells were trypsinized, seeded at a density of 5 × 10^3^ cells/well into 96-well plates (Corning, Tewksbury, MA, USA) in quadruplicate, and left to adhere for the entire night. The culture medium was changed the following day to an estrogen-free medium that had been supplemented with 5% FBS for a whole day. Next, using PBS as a control, the cells were treated with ACh, E2, and Ful, following the protocol. Following treatment, the cells were incubated for 4 h at 37 °C with 10% MTT in the hormone-reduced medium. After that, the medium was carefully removed and the formazan precipitate was dissolved by adding 100 μL of DMSO. A microplate reader (PHOmo, Autobio Labtec Instruments, Zhengzhou, China) was used to measure the absorbance at 492 nm. The ratio of the absorbance in wells under PBS treatment was used to calculate the viability rate. Each experiment was carried out four times.

### 2.5. Nuclear Protein Isolation

Thermo Fisher Scientific, Waltham, MA, USA, provided the NE-PER^TM^ Nuclear and Cytoplasmic Extraction Reagents for the extraction of nuclear proteins, as described before [45].

### 2.6. Western Blot

Western blotting was carried out to determine protein levels, as previously described [45]. The cells were seeded to 80% confluence in 60 mm dishes and lysed on ice, using RIPA lysis buffer (pH 7.4, 0.5 M Tris-HCl, 1.5 M NaCl 2.5%, deoxycholic acid, 10% NP-40, and 10 mM EDTA). A protease inhibitor cocktail (Cell Signaling Technology, Danvers, MA, USA) was added and centrifuged for 15× *g* min at 4 °C at 13,200 rpm. Using the PierceTM kit, the number of cellular proteins in the supernatant was measured, following the manufacturer’s instructions. Following quantification, 20 µg of protein was dissolved in a loading buffer that contained 60 mM Tris (pH 6.5, 10% (*v*/*v*)), 10% (*v*/*v*), glycerol, 0.025% (*w*/*v*) bromophenol blue, and 20% (*w*/*v*) SDS β-mercaptoethanol. The protein was then denatured for five minutes at 95 °C. After the SDS-PAGE on 12.5% acrylamide gels in a mini protean tetra cell (Bio-Rad Laboratories, Inc., Hercules, CA, USA) was carried out, the gels were electroblotted onto PVDF membranes (Amersham^TM^ Hybond^®^, Marlborough, UK) using a Trans-Blot^®^ Semi-Dry Transfer Cell (Bio-Rad Laboratories, Inc.) at 20 V for 50 min. To block the membranes, a blocking solution consisting of 5% bovine serum albumin dissolved in Tris-buffered saline (TBS); 1% Tween 20 (TBS-T20) at room temperature was used for two hours. The membranes were then incubated overnight at 4 °C with a primary antibody dissolved in the blocking solution, after being washed three times for 15 min each with TBS-T20. The antibodies used were anti-ERα (D6R2W), p-Akt^S473^ (D9E), and Akt-pan (C67E7) from Cell-Signaling Technology (Danvers, MA, USA); M1 (ab77098), M2 (ab2805), M3 (ab126168), p-ERαS^118^ (ab32396), p-ERα^S104/106^ (ab269451), p-ERα^S167^ (ab31478), and p-ERK1^T202^ (ab194776) from Abcam (San Francisco, CA, USA); and ß-Actin (sc-47778), ERK1 (sc-271291), PCNA (sc-25280), Lamin B1 (sc-377000), ERK 1/2 (sc-514302) from Santa Cruz Biotechnology Inc. (Santa Cruz, CA, USA). The membranes were treated for 2 h with secondary anti-rabbit or anti-mouse peroxidase-conjugated antibodies that were diluted to 1:2000 and dissolved in a blocking solution. Following incubation, chemiluminescent HRP substrate (Clarity Western ECL cat no. 1705060) was applied to the protein bands of the membranes, which allowed them to be seen using a chemiluminescence scanner (C-DiGit^®^, LIQUOR Biosciences, St. Louis, MO, USA). Using Image J software 1.53t (Wayne Rasband, NIH), quantitative assessments of the band-density data were carried out, using ß-Actin as a loading control.

### 2.7. Immunofluorescence and Confocal Microscopy

To determine the subcellular localization of ERα, indirect immunofluorescence (IF) was used, as previously described [45]. The MCF7 cells were cultured in chamber slides (Nunc Inc., Naperville, IL, USA) until confluence. After that, the cells were dried, washed twice with PBS (pH 7.2), and fixed for ten minutes in cold methanol (MetOH). Following three PBS washes, they were permeabilized with 0.1% Triton X-100 and blocked for one hour at room temperature with 3% bovine serum albumin (BSA) (Rockland Immuno-chemicals, Inc., Limerick, PA, USA). The anti-ERα monoclonal antibody (sc-8002) (Santa Cruz Biotechnology, Dallas, TX, USA) was then diluted in PBS 1% BSA and incubated overnight at 4 °C (1:200 dilution) in the chambers. The next day, the cells were incubated with a secondary fluorescein isothiocyanate antibody (FITC) from Santa Cruz Biotechnology Inc. (Dallas, TX, USA) for two hours at room temperature, after being washed three times for fifteen minutes at room temperature. Following three PBS washes, the cells were incubated at room temperature for 15 min with DAPI (1:10,000) (Thermo Fisher Scientific, Waltham, MA, USA), and the results were observed using a C2 Plus confocal microscope.

### 2.8. Immunocytochemistry (ICC)

The cells were plated at a density of 1 × 10^4^ cells/mL on a glass chamber slide and grown to 80% confluence, as previously described [47]. Protein expression was evaluated by peroxidase immunochemical staining. Exponentially growing control cells and irradiated cells were plated on a four-well glass chamber slide (Nunc Inc., Naperville, IL, USA). The cells were allowed to grow at a density of 1 × 10^4^ cells in 1 mL of medium for 2–3 days until they reached 70% confluency. The cells were incubated with 1% H_2_O_2_ in methanol for 30 min to block endogenous peroxidase, washed twice with a buffer solution, and fixed with buffered paraformaldehyde at 5% in PBS, pH 7.4, at room temperature. Subsequently, the cell cultures were covered with normal horse serum for 30 min at room temperature. The cultures were then washed once and incubated with the corresponding antibodies at a 1:500 dilution overnight at 4 °C. The cells were then treated with mouse monoclonal primary antibodies directed against PCNA (sc-56, Santa Cruz Biotechnology Inc., Santa Cruz, CA, USA); M1 (ab77098), M2 (ab1805), and M3 (ab150480) (Abcam, San Francisco, CA, USA); and ERα (sc-8002, Santa Cruz Biotechnology, Inc., Santa Cruz, CA, USA). These primary antibodies were employed at a 1:500 dilution and incubated at 4 °C for an entire night. After two PBS washes, the cells were treated for 45 min with diluted biotinylated secondary antibody solution from Vector Laboratories Inc. in Burlingame, California, and Vectastin Elite ABC reagent. By contrasting the staining intensity of the treated cells with that of the untreated control cells, a semi-quantitative calculation, based on the relative staining intensity of protein expression, was established. Images were captured using an Olympus CX31 optical microscope (40×).

### 2.9. Ca^+2^ Influx Assay

Cellular Ca^+2^ concentrations were quantified using the fluorescent dye Fluo-4 (Thermo Fischer Scientific, Waltham, MA, USA), according to the protocol suggested by Gee et al. [48]. Cells at approximately 80% confluence were seeded onto a glass-bottomed dish and incubated for 30 min with DMEM medium that lacked calcium and contained the Fluo-4 probe. After that, ACh was applied to the cells for 10 min, and a C2 Plus confocal microscope was used to capture Fluo-4 fluorescence images at 488 nm every five seconds. Using ImageJ Fiji software (version 1.52b, NIH), the Fluo-4 fluorescence signal in each cell was examined. The relative fluorescence F/F0, where F and F0 stand for the fluorescence intensity at each time point and the initial fluorescence value (at 0 s), respectively, was used to express changes in intracellular Ca^+2^ levels.

### 2.10. Protein–Protein Interaction Networks

Functional enrichment analysis of protein–protein interaction was carried out by STRING, a Web Core Data Resource, version 11.5 (https://string-db.org/), accessed on 22 March 2023.

### 2.11. Statistical Analysis

The data were expressed as the mean ± standard error. ANOVA and the Student’s *t*-test were used to examine the statistical significance between the untreated and treated groups. Statistical significance was defined as *p* < 0.05. At least three independent assays were conducted. For statistical analysis, GraphPad Prism version 5.0 software (GraphPad Software, Inc., La Jolla, CA, USA) was used.

## 3. Results

### 3.1. Cell Viability

**Figure 1:**
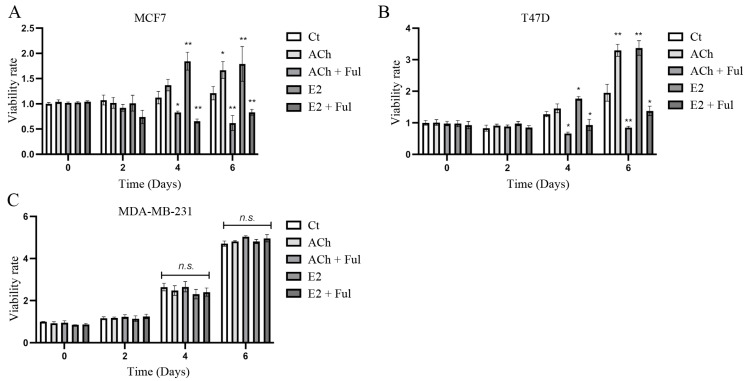
Viability rate analyzed by MTT assay in (**A**) MCF7, (**B**) T47D, and (**C**) MDA-MB-231 cell lines after 2, 4, and 6 days of incubation in complete media. Effect of ACh (1 × 10^−7^ M) and E2 (1 × 10^−8^ M) in the absence or presence of Fulvestrant (Ful) at 10 nM for 24 h. DMSO was added to the control wells in an equivalent concentration. Asterisks denote statistically significant differences in comparison with vehicle-treated cells. *: *p* < 0.05; **: *p* < 0.01. n.s: non-significant.

Cell viability rate was evaluated by MTT assays to determine the effect of E2 and ACh in the presence or absence of Ful, a selective ER degrader (SERD), in MCF7, T47D, and MDA-MB-231 cell lines for 2, 4, and 6 days, as shown in Figure 1A–C. The results indicated that both E2 and ACh induced a significant (*p* < 0.01) increase in cell viability in MCF7 and T47D cell lines after 4 and 6 days of treatment, and such effect was decreased by 10 mM of Ful. However, the results showed that the MDA-MB-231 cell line did not follow the same pattern. Thus, these results suggested that cell viability induced by ACh was mediated in an ERα-dependent manner.

### 3.2. Proliferating Nuclear Antigen (PCNA), Muscarinic Receptors M1, M2, M3, and Estrogen Receptor Alpha (ERα) Protein Expression in Breast Cell Lines

**Figure 2:**
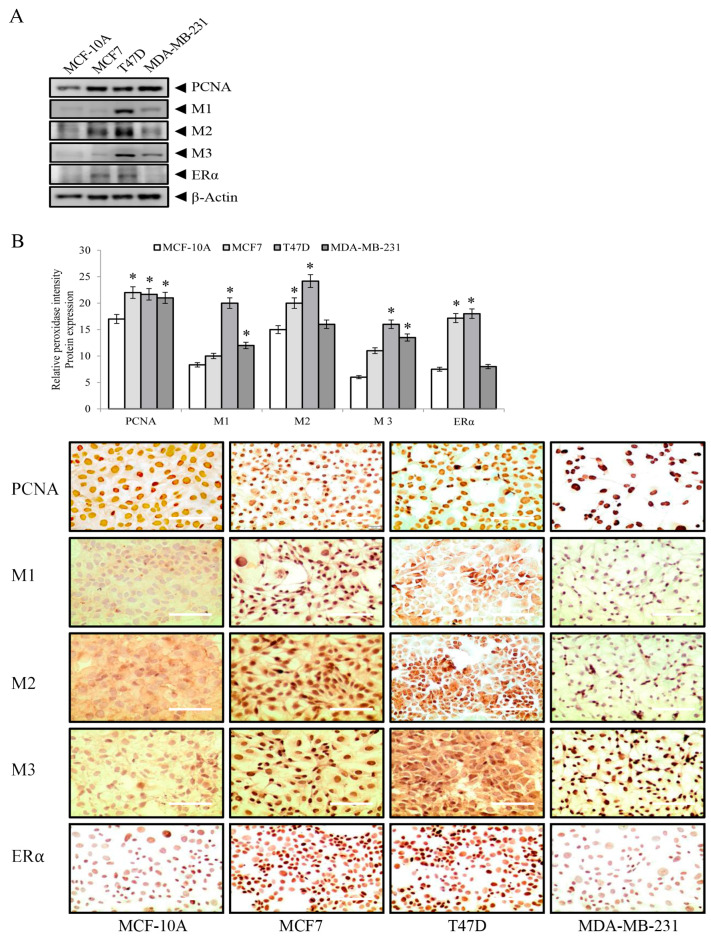
Proliferating nuclear antigen (PCNA), muscarinic receptors M1, M2, M3, and estrogen receptor alpha (ERα) protein expression in MCF-10A, MCF7, T47D, and MDA-MB-231 cell lines by (**A**) Western blot, β-Actin the loading control; (**B**) graph that represents PCNA, M1, M2, M3, and ERα protein expression by relative peroxidase intensity in MCF-10A, MCF7, T47D, and MDA-MB-231 cell lines and their corresponding representative peroxidase images of immunochemical studies of PCNA (sc-56, Santa Cruz Biotechnology Inc., Santa Cruz, CA, USA); M1, M2, and M3 (ab77098, ab1805, ab150480, respectively) (Abcam, San Francisco, CA, USA); and ERα (sc-8002, Santa Cruz Biotechnology Inc., Santa Cruz, CA, USA) in MCF-10A, MCF7, T47D, and MDA-MB-231 cell lines. Images were taken with an Olympus optical microscope CX31, scale bar 100 µm (40× magnification). *: *p* < 0.05.

Western blot analysis revealed a significant increase (*p* < 0.05) in PCNA levels (Figure 2A), indicating enhanced cell proliferation in the MCF7, T47D, and MDA-MB-231 cell lines after 4 days of incubation, compared to control MCF-10A cells. Additionally, M1, M2, and M3 protein-expression patterns exhibited distinct cell-line-specific variations, with T47D and MDA-MB-231 expressing higher levels than those of MCF-10A and MCF7. Similarly, ERα protein expression was significantly higher in MCF7 and T47D than in MCF-10A and MDA-MB-231 cell lines. Immunochemical studies (Figure 2B) further supported the Western blot findings, demonstrating the distinct protein expression profiles observed in the different cell lines. Optical density charts for Figure 2 can be found in Figure S1.

**Figure 3:**
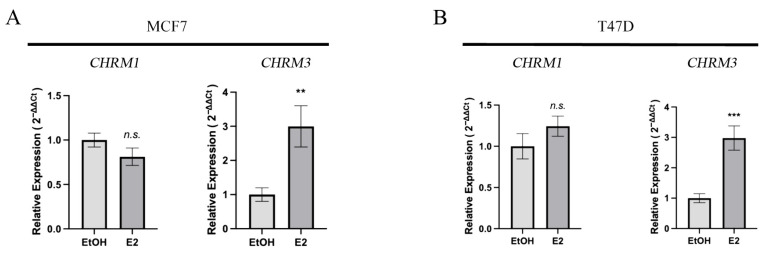
Determination of the effect of 17β-estradiol (E2) on muscarinic receptor genes *CHRM1* and *CHRM3* expression levels in (**A**) MCF7, and (**B**) T47D cell lines. Asterisks denote statistically significant differences in comparison with vehicle-treated cells. **: *p* < 0.01; ***: *p* < 0.001, n.s.: non-statistically significant.

The present study also analyzed the effect of E2 on muscarinic receptor gene expression in MCF7 (Figure 3A) and T47D cell lines (Figure 3B). The results of such studies indicated that the expression level of the cholinergic receptor muscarinic 1 gene, *CHRM1*, was not significant in either the MCF7 or the T47D cell line in comparison with their controls, containing only the vehicle. However, the cholinergic receptor muscarinic 3 gene, *CHRM3*, was significantly higher in MCF7 and T47D (*p* < 0.01 and *p* < 0.001, respectively) than in the control containing only the vehicle.

### 3.3. Acetylcholine and Calcium

**Figure 4:**
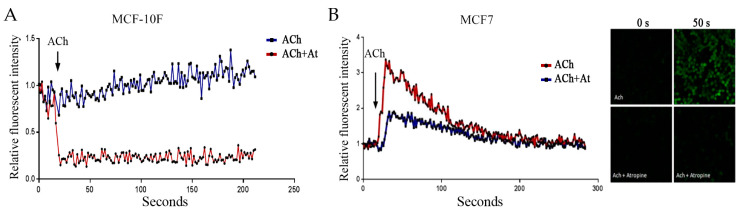
Effect of acetylcholine (ACh) (1 × 10^−7^ M) and atropine (At) (5 μM) on fluorescence intensity in (**A**) the MCF-10F cell line and (**B**) the MCF7 cell line, provided by Fluo-4 AM fluorophore, demonstrating the release of Ca^+2^ over time. Representative images of Ct, ACh, ACh + atropine at 0 s and 50 s in MCF7. Images were taken with a Nikon C2 Plus confocal microscope.

To assess ACh functionality in BC cells, intracellular Ca^+2^ levels were measured through the green fluorescent calcium indicator Fluo-4. Figure 4A shows that intracellular Ca^+2^ levels slightly increased in time after being exposed to ACh in the MCF-10F cell line, whereas exposure to a combination of both ACh and 10μM of atropine, a selective mAChRs antagonist, decreased those levels after a few seconds, maintaining a steady phase, whereas in Figure 4B the intracellular Ca^+2^ levels increased fluorescent probe signal, showing a peak at approximately 40 s—then, the intensity fell to values close to pre-stimulation, around 200 s. Representative images of Ct, ACh, ACh + atropine at 0 s and 50 s in MCF7 are shown in the right panel of Figure 4B. These results indicated that ER-negative cells (MCF-10F) did not show intracellular calcium mobilization in response to Ach. This effect was contrary to what occurred in MCF7 (a positive Erα cell line), where ACh increased intracellular calcium, an effect inhibited by atropine.

### 3.4. Acetylcholine Leads to Phosphorylation of Akt and ERK 1/2 in Breast Cell Lines

**Figure 5:**
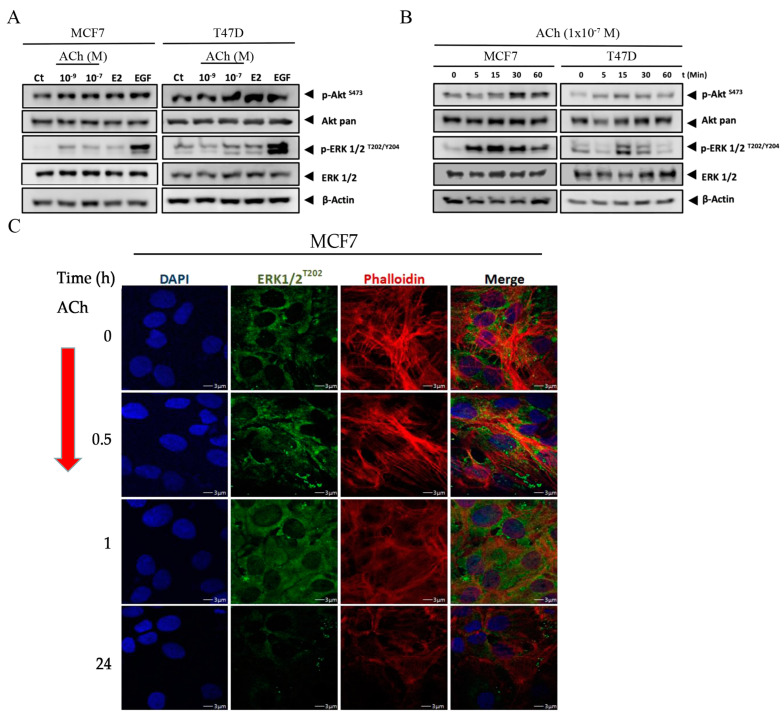
(**A**) Acetylcholine (ACh) on dose kinetic of MCF7 and T47D cell lines at 1 × 10^−7^ M and 1 × 10^−9^ M by Western blot; 17β-estradiol (E2) was used at 1 × 10^−8^ M and EGF at 1 nM. (**B**) Effect of ACh at 1 × 10^−7^ M on time kinetic after 30 min; 17β-estradiol (E2) at 1 × 10^−8^ M and EGF at 1 nM were considered in these cell lines. These studies were carried out by Western blot on the activity of the PI3K and MAPK pathways, assessed by measuring levels of phosphorylation of pAkt^Serine743^ and pERK1/2^T202/Y204^. Antibodies used were Akt-pan (C67E7, Cell-Signaling Technology, Danvers, MA, USA), ERK1/2 (sc-514302) and ERK1 (sc-271291) (Santa Cruz Biotechnology Inc., Santa Cruz, CA, USA), and p-ERK1/2^T202/Y204^ (ab194776) (Abcam, San Francisco, CA, USA). β-Lamin (sc-377000) (Santa Cruz Biotechnology Inc., Santa Cruz, CA, USA) was the nuclear control and β-Actin (sc-47778) (Santa Cruz Biotechnology Inc., Santa Cruz, CA, USA) was the loading control. (**C**) ACh on ERK1/2^T202/Y204^ phosphorylation after 30 min, 1 h, and 24 h. DAPI is a fluorescent marker of DNA as a nuclear stain in blue color. Phalloidin is a filament-cell indicator in red color. The fourth column to the right indicates the merger of two previous colors and the colocalization of previous proteins. Images are representative of three independent experiments. The images were taken with a Nikon C2 Plus confocal microscope.

To elucidate the signaling pathways triggered by ACh in breast cancer cell lines, we assessed the activation of the MAPK and PI3K pathways by quantifying the phosphorylation levels of ERK and Akt, respectively. This analysis was conducted using commercially available antibodies designed to detect the phosphorylated forms of ERK and Akt. The results demonstrated a notable increase in Akt^S473^ phosphorylation induced by ACh, E2, and EGF when compared to the control in both the MCF7 and T47D cell lines. This observation indicates the activation of the PI3K/Akt pathway (Figure 5A). Similarly, the phosphorylation of ERK½ at T202/Y204 residues exhibited an increase in response to ACh, E2, and EGF in both cell lines, compared to the control (Figure 5A). These findings provide evidence for the activation of the MAPK pathway. Collectively, these results demonstrate the activation of both the PI3K/Akt and MAPK/ERK pathways in ERα-dependent breast cancer cell lines in response to Ach.

To further characterize the kinetics by which MAPK is activated by ACh, MCF7 and T47D cells were exposed to 1 × 10^−7^ M of ACh over a wide range of times and the phosphoproteins levels were evaluated by Western blot (Figure 5B). The results indicate that both ERK and p38 were phosphorylated after 5 min of treatment, while PI3K was activated at 30 and 15 min after treatment in the MCF7 and T47D cell lines respectively, which agreed with the quick response observed previously for the Ca^+2^ release. In summary, these results collectively suggest that ACh triggers the activation of both the PI3K and MAPK signaling pathways in breast cancer cell lines, as evidenced by the increased phosphorylation of Akt and ERK1/2, particularly after 30 min of exposure. Figure 5C shows the effect of ACh (1 × 10^−7^ M) on p-ERK^T202/Y204^ in the MCF7 cell line after 30 min, 1, and 24 h, analyzed by confocal microscopy using a secondary antibody conjugated to the FITC fluorophore. Representative images indicated that the phosphorylation occurred after 30 min due to the effect of Ach, but it disappeared after 24 h. Optical density charts for Figure 5 can be found in Figure S2.

### 3.5. Acetylcholine and Estrogen Induction of ERα Phosphorylation and Nuclear Recruitment in MCF7 Cell Line

**Figure 6:**
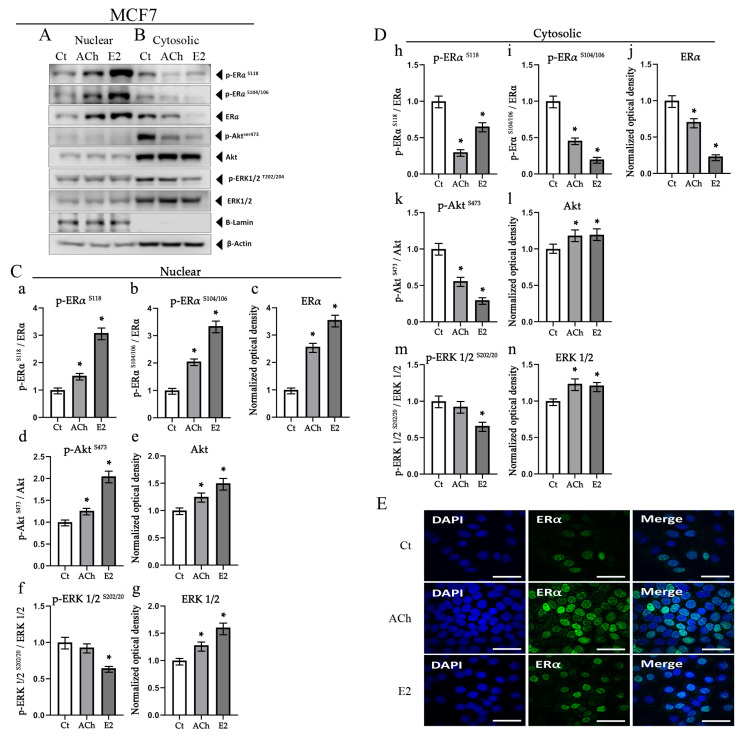
Effect of acetylcholine (ACh) (1 × 10^−7^ M) and 17b-estradiol (E2) (1 × 10^−8^ M) on the phosphorylated forms of (**A**) the nuclear portion and (**B**) the cytosolic portion in the MCF7 cell line by Western blot. Bars (**Ca**–**Cg**) represent the nuclear recruitment experiments and bars (**Dh**–**Dn**) represent the cytosolic experiments in the MCF7 cell line after 30 min by Western blot. Nuclear and cytosolic lysates were obtained for Western blot analysis. Commercial antibodies were used to detect the phosphorylated form of ERα at serine 104/106 and 118 in the entire lysates of the cell lines subjected to ACh and E2. It can be seen as representative Westerns of three independent experiments. (**E**) Representative images of the effect of ACh and E2 on ERα in MCF7 cells. The secondary antibody employed was coupled to the fluorescent DNA marker DAPI and the fluorophore FITC. Images are representative of three independent experiments and were taken with a Nikon C2 Plus confocal microscope. *: *p* < 0.05.

Then, we assessed whether ERα phosphorylation induced by ACh and E2 was associated with a rise in its nuclear recruitment in MCF7 (Figure 6). The findings showed that E2 exposure caused ERα and its phosphorylated forms to be recruited into the nucleus in MCF7. ACh also triggered the activation of the Akt and ERK1/2 signaling pathways. It was observed that such pathways were found to operate concurrently with the phosphorylation of ERα and its subsequent translocation into the nucleus. The nuclear fractions can be seen in Figure 6(Ca–Cg), where the bars correspond to the effect of ACh at 1 × 10^−7^ M and E2 at 1 × 10^−8^ M on ERα ^S118^; pERα ^S104/106^; ERα; Akt^S473^; Akt; ERK½ ^T202/Y204^; and ERK½ protein expression. Graphs of the cytosolic fractions can be seen in Figure 6(Dh–Dn), where the bars correspond to the effect of ACh at 1 × 10^−7^ M and E2 at 1 × 10^−8^ M on total ERα, Akt, and ERK1/2 protein expression and its phosphorylated forms. Thus, ACh (1 × 10^−7^ M) and E2 (1 × 10^−8^ M) also triggered the activation of the PI3K and MAPK signaling pathways after 30 min, in comparison with Ct. These results showed that ACh and E2 increased the expression intensity of ERα in the nucleus, being ACh effect higher than that of E2, as a merger by colocalization, as shown in Figure 6E. The representative images depict immunofluorescence through confocal microscopy, where a secondary antibody was coupled to the FITC fluorophore and the DAPI.

**Figure 7:**
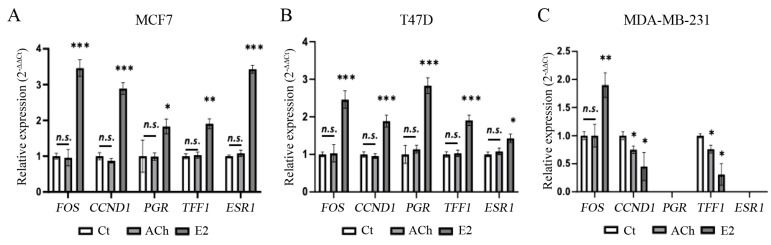
Effect of acetylcholine (ACh) (1 × 10^−7^ M), 17β-estradiol E2 (1 × 10^−8^ M), or vehicle during 4 h on c-fos, *FOS*, and *CCND1* (cyclin D1); progesterone receptor, *PGR*; trefoil factor 1 (pS2), *TFF1* and *ESR1* (ER-alpha) gene expression in (**A**) MCF7, (**B**) T47D, and (**C**) MDA-MB-231 cell lines by q-RT-PCR. The transcript levels were compared to the expression of the ß-Actin gene. Values represent the means ± SEM of three separate studies, each carried out in triplicate. Asterisks denote statistically significant differences in comparison with vehicle-treated cells. *: *p* < 0.05; **: *p* < 0.01; ***: *p* < 0.001; n.s.: non-statistically significant.

To evaluate whether ACh triggers estrogenic gene expression, we evaluated the gene expression of well-known estrogen-responsive genes. Figure 7 shows genes such as *FOS* (c-fos), *CCND1* (cyclin D1), *PGR* (progesterone receptor), *TFF1* (trefoil factor 1, pS2); and *ESR1* (ERα) in (A) MCF7, (B) T47D, and (C) MDA-MB-231 cell lines after 4 h of treatment. The results show that E2 treatment significantly increased *FOS*, *CCND1*, *ESR1* (*p* < 0.001), *TFF1*(*p* < 0.01), and *PGR* (*p* < 0.05) gene expression analyzed in the MCF7 cell line. E2 also significantly increased *FOS*, *CCND1*, *PGR*, *TFF1* (*p* < 0.001), and *ESR1* (*p* < 0.05) in the T47D cell line. E2 significantly (*p* < 0.01) increased FOS; however, it significantly (*p* < 0.05) decreased *CCND1* and *TFF1* in the MDA-MB-231 cell line. ACh at 1 × 10^−7^ M did not induce a statistically significant increase in the genes analyzed in the MCF7 and T47D cell lines, except for a significant (*p* < 0.05) decrease in CCND1 and *TFF1* in the MDA-MB-231 cell line. Consequently, ACh failed to induce the overexpression of estrogen-responsive genes, suggesting a different mechanism of activation than 17β-estradiol.

### 3.6. Acetylcholine and Estrogen on Epithelial–Mesenchymal Transition Gene Expression in Breast Cell Lines

**Figure 8:**
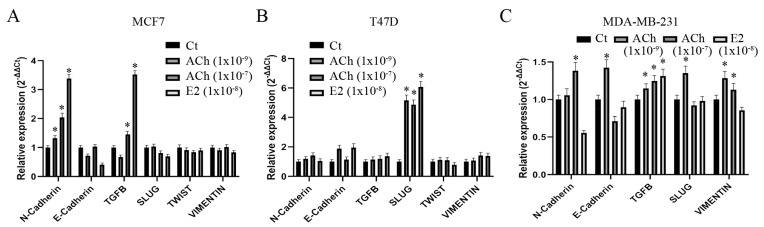
Effect of ACh (1 × 10^−9^ M and 1 × 10^−7^ M) and 17β-estradiol (E2) (1 × 10^−8^ M) by q-RT-PCR on several genes related to epithelial–mesenchymal transition such as *N-Cadherin*, *E-Cadherin*, *TGFB*, *SLUG*, *TWIST*, and *VIMENTIN* expression levels in (**A**) MCF7, (**B**) T47D, and (**C**) MDA-MB-231 cell lines. *: *p* < 0.05.

Studies were carried out to confirm the relative gene-expression alterations induced by ACh and E2 on EMT-related genes in the MCF7, T47D, and MDA-MB-231 cell lines by q-RT-PCR. Figure 8A shows that ACh at 1 × 10^−9^ M and 1 × 10^−7^ M and E2 (1 × 10^−8^ M) significantly (*p* < 0.05) increased *N-Cadherin* gene expression in the MCF7 cell line, while ACh at 1 × 10^−7^ M and E2 increased *TGFB* expression levels. There was no effect on *E-Cadherin*, *SLUG*, *TWIST*, and *VIMENTIN* in this cell line. Furthermore, Figure 8B indicates that ACh at 1 × 10^−9^ M and 1 × 10^−7^ M and E2 significantly (*p* < 0.05) increased *SLUG* gene expression in the T47D cell line.

In Figure 8C, it can be seen that ACh at 1 × 10^−7^ M significantly (*p* < 0.05) increased *N-Cadherin* in MDA-MB231; ACh at 1 × 10^−9^ M increased *E-Cadherin* in the same cell line; ACh at 1 × 10^−9^ M and 1 × 10^−7^ M and E2 (1 × 10^−8^ M) significantly (*p* < 0.05) increased *TGFB*; and ACh increased *SLUG* at 1 × 10^−9^ M and *VIMENTIN* at 1 × 10^−9^ and 1 × 10^−7^ M in the same cell line. ACh and estrogen increased *N-Cadherin* and *TGFB* gene expression in the MCF7 and *SLUG* in the T47D cell line. ACh alone increased *N-Cadherin*, *E-Cadherin*, *SLUG*, and *VIMENTIN* in MDA-MB-231, while either ACh or E2 increased *TGFB* in the same cell line, leading to gene alteration.

**Figure 9:**
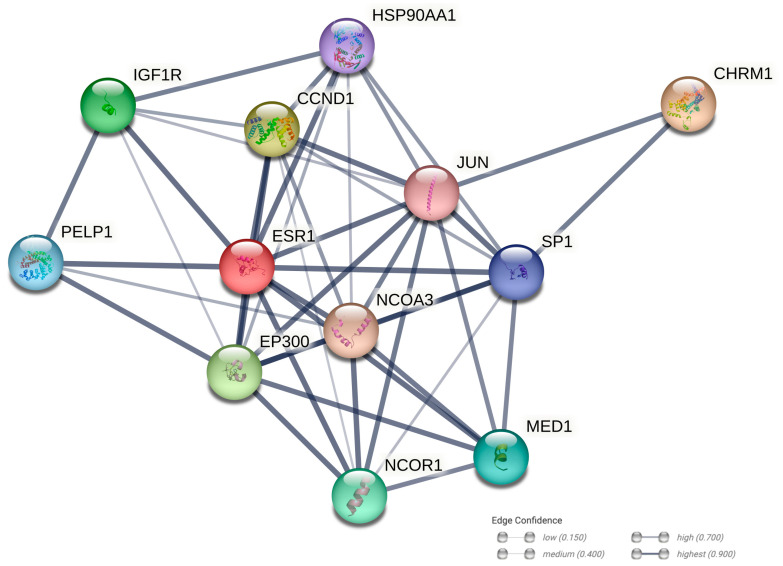
Protein network analysis carried out by STRING analysis. The network shows the interactions between estrogen receptor-alpha (ESR1) and muscarinic receptor 1 (CHRM1). The interaction network is made up of nodes and edges that stand for proteins and functional/physical connections, respectively. Line thickness indicates the strength of data support. The analysis was carried out with medium confidence (0.400) and considering 10 interactors for the first shell.

To elucidate a hypothetical interaction between the estrogen ERα and CHRM1, we employed a string analysis from the STRING web resource. As shown in Figure 9, this analysis suggested a functional interaction between them through the transcription factors c-Jun and Sp1. The STRING analysis indicated a moderate confidence score for the interaction, based on evidence from curated databases and experimentally determined protein–protein interactions. This proposed interaction could be pivotal in modulating signaling pathways that are crucial in both neuroendocrine regulation and the progression of breast cancer, suggesting a complex interplay that might contribute to the disease’s pathophysiology and which could, potentially, open new avenues for therapeutic intervention.

## 4. Discussion

Previous data from our group showed that ACh serum levels increased at the same time as mammary tumor formation in rats exposed to OPs [11,12,13,14]. Subsequently, other authors demonstrated that mAChR activation can stimulate cell proliferation in estrogen-dependent cells [34,35]. Therefore, here, we propose that signaling pathways activated by mAChR in response to ACh exposure may promote ERα activity, with consequences on cell viability in BC cell lines. Although the molecular mechanism by which ERα activity is not completely solved, we hypothesized that ERα activation can occur by a ligand-independent mechanism through MAPK-ERK pathway activation.

First, to assess the physiological relevance of our study, we analyzed the presence of mAChRs in surgically obtained biopsy samples of breast cancer patients. According to the literature, a large body of evidence has documented a wide mAChR distribution throughout the central and peripheral nervous systems [49] and several tissues, like smooth muscle [50], the gastrointestinal tract [51], the urinary bladder [52], the heart [53], the lungs [54], the eye [55], and blood vessels [56], with numerous physiological functions have been studied. However, studies on the role of mAChRs in mammary tissue are limited. Lombardi et al. (2013) documented that the M2 and M3 subtypes are overexpressed in samples from patients with breast tumors in different grades [29]. Additionally, another study demonstrated that murine breast adenocarcinoma cell lines LM3 and LM2 expressed all mAChR subtypes [57]. Consistent with these data, our results revealed differential expression patterns of M1, M2, and M3 receptor subtypes among the evaluated breast cancer cell lines. Our immunohistochemical analysis demonstrated the overexpression of M2 and M3 in cases of dysplasia and ductal carcinomas. These results suggest a conserved role of mAChRs in cancer cells, hinting at their involvement in fundamental cellular processes and signaling pathways that contribute to the intricate biology of breast tumors. Similarly, the variations in mAChR subtype expression may be associated with the complex nature of breast tumors, characterized by widespread differential mRNA expression and frequent mutation rates [58].

An interesting aspect of BCs is the fact that approximately 70–75% of them overexpress ERα, indicating a high level of estrogen dependence for cell growth. Interestingly, the colocalization of ERα and mAChRs on neurons in the central nervous system has provided evidence of an interaction of steroid hormone receptors and mAChRs [58]. Additionally, Cardoso et al. (2004) showed that E2 modulates the expression and function of mAChRs in the rat hippocampus [59]. In the uterus, mAChR expression is also influenced by the hormonal environment and E2 induces an increase in myometrial responsiveness to mAChR agonists [44]. Therefore, we evaluated the possibility that *CHRM* expression levels were dependent on estrogen supply and ERα activity in BC cells. As shown in Figure 4, mRNA levels of the M3 mAChR increased with E2 treatment for 4 h. However, these effects were not observed in the ERα (−) cell line MDA-MB-231 or in those transfected cells, which indicates the need for other additional factors characteristic of lines sensitive to estrogens. These results show that, like hippocampal and uterine cells, circulating estrogen levels could modulate mAChRs expression in BC cells, which could have profound implications for the origin and progression of cancer cells at the physiological level. For example, the sensitivity of BC cells to ACh or some type of neuromodulator could have a high variability during pregnancy, with consequences for the aggressiveness and progression of the cancer. Similarly, under chronic stress, it has been reported that the continuous release of neurotransmitters from the neuroendocrine system has a highly profound impact on the occurrence and prognosis of BC [60]. Therefore, the mAChR activity influenced by E2 could be part of a molecular mechanism through which chronic stress promotes BC via neurotransmitters secreted by the nervous system.

MAChRs exert their functions through second messenger cascades by coupling to specific classes of G-proteins. Here, we demonstrated that ACh exposure led to quick Ca^+2^ release, which was blocked under atropine pre-treatment in MCF7 cells, suggesting that that was a specifically muscarinic effect through M1, M3, or M5 subtypes. Next, we observed that MAPK/ERK and PI3K signaling pathways are activated concomitantly with Ca^+2^; therefore, it could be hypothesized to be a Ca^+2^-dependent mechanism. However, to corroborate this assumption, experiments of phosphorylation of MAPK/ERK induced by ACh in a Ca^+2^ free medium by adding chelators would be a good approach. In the same line, we cannot rule out the role of M2 and M4, since MCF7 and T47D cells maintain basal levels of them.

As documented in the literature, it is well-established that ERα activation can primarily arise from the phosphorylation of specific receptor residues or from interactions with coregulators [61]. Consequently, to assess the potential ligand-independent activation of ERα by ACh, we conducted measurements of the phosphorylation status and subcellular localization of ERα. In this regard, our results demonstrated an increase in the phosphorylated form of ERα, accompanied by its translocation into the nucleus after ACh treatment. This finding suggests an enhancement of ER activity upon activation of mAChRs, indicating a possible crosstalk between mAChRs signaling triggered by ACh and ERα phosphorylation.

ERs are DNA-binding transcription factors that are ligand-modified to regulate the gene expression of certain genes in response to estrogens [62]. To assess whether ACh imitates ERα transcriptional activity akin to that of E2, we evaluated the expression levels of well-known estrogen-responsive genes. Our findings revealed that although ERα phosphorylation was enhanced by ACh treatment, this did not elicit a transcriptional response in some estrogen-regulated genes. This outcome aligned with our expectations, considering the distinct mechanisms of activation involved in the ACh and E2 signaling pathways. It is worth noting that the non-genomic effects of ERα may involve additional transcription factors that were not evaluated in this study.

Indeed, our analysis, utilizing a web resource for protein–protein associations, revealed a functional interaction between ESR1 and CHRM1, mediated by the transcription factors c-Jun and SP1 (Figure 9). Notably, these factors were not investigated in our current study. Thus, it is plausible that c-Jun and SP1 may play crucial roles in mediating the transcriptional activity of Erα, induced by the activation of mAChRs.

To further investigate the impact of ACh and E2 on epithelial–mesenchymal transition (EMT), we conducted studies of three breast cancer cell lines: MCF7, T47D, and MDA-MB-231. These investigations aimed to confirm the relative gene-expression alterations induced by these substances and to shed light on their implications. In the MCF7 cell line, the results demonstrated that both ACh and E2 led to an increase in the expression of N-Cadherin and TGFB genes. This suggests that the activation of muscarinic receptors by ACh and the action of E2 may promote EMT in ER-positive breast cancer cells, potentially contributing to the invasive and metastatic characteristics associated with this transition. Conversely, in the T47D cell line, the substances exhibited a different pattern of gene-expression alteration. Here, ACh and E2 specifically increased the expression of the SLUG gene. This differential response between MCF7 and T47D cells underscores the complexity of the interplay between the ACh, E2, and EMT pathways in breast cancer, even within ER-positive subtypes. The most intriguing findings emerged from the MDA-MB-231 cell line, characterized as ER-negative. In this cell line, ACh uniquely increased the expression of N-Cadherin and E-Cadherin, SLUG, and VIMENTIN genes, whereas both ACh and E2 elevated TGFB gene expression. These results suggest that in ER-negative breast cancer cells, ACh might play a role in orchestrating a more complex modulation of EMT-related genes. Moreover, the observed increase in TGFB expression further emphasizes the resistance of ER-negative patients to hormone therapy.

In summary, our results show that ACh promotes breast cancer cell proliferation and ERα activity, possibly in a ligand-independent manner through the MAPK/ERK signaling pathway, with consequences for the expression of EMT markers, suggesting its putative role in breast cancer progression.

Despite describing the effects of ACh on breast cancer cell lines, our study has several limitations. First, the study model was largely limited. The results rely heavily on specific cell lines (MCF7, T47D, and MDA-MB-231), which represent certain breast cancer subtypes but do not encapsulate the full diversity and heterogeneity of breast tumors. This could limit the generalizability of the findings to other breast cancer types and in vivo settings, where factors like immune responses, blood supply, and tissue architecture can influence outcomes. Second, while the study demonstrated various effects, such as increased cell viability in certain conditions, the precise molecular mechanisms behind these phenomena were not fully elucidated. Finally, the exposure of cells to multiple substances (e.g., ACh, E2, and Ful) could introduce confounding variables, making it difficult to attribute observed effects solely to one particular substance.

The implications of these findings are significant, revealing potential new pathways for therapeutic intervention and a deeper understanding of breast cancer pathophysiology. Building upon these results, several avenues for future research could emerge. First, the differential expression of M1, M2, and M3 mAChRs in various breast cancer cell lines suggests subtype-specific roles. Therefore, future studies should expand this analysis to a broader range of breast cancer subtypes, including those with different hormonal statuses and genetic backgrounds. Clinical studies are crucial to validate these findings in humans, and patient-derived data, including responses to treatments targeting muscarinic receptors, would be valuable. These studies could also explore the potential prognostic value of muscarinic receptor expression levels in breast cancer.

Another important area for future research is the cholinergic autocrine cycle in cancer cells. Future studies could focus on the synthesis, release, and autocrine/paracrine actions of ACh in breast cancer cells and investigate how these mechanisms affect interactions with the tumor microenvironment. Additionally, while this study highlighted ERα-dependent pathways, the possibility that ACh influences hormone-independent breast cancer cells cannot be ruled out. New insights from this research could inform treatment strategies for ER-negative breast cancers.

Finally, given the study’s implication of a link between chronic stress, the neuroendocrine system, and breast cancer, longitudinal studies to explore this relationship further would be worthwhile. Such studies could involve monitoring stress levels and neuroendocrine system markers in patients with breast cancer over time. These proposed future studies would offer a better understanding of breast cancer pathophysiology.

## 5. Conclusions

Our findings demonstrated that ACh stimulated breast cancer cell proliferation and enhanced the activity of ERα, potentially through a ligand-independent mechanism mediated by the MAPK/ERK signaling pathway. These alterations in ERα activity have downstream effects on the expression of EMT markers, implying a potential role for ACh in driving breast cancer progression. Therefore, this study proposes that ACh and muscarinic receptors seem to be involved as emerging regulators of the carcinogenesis process and opens a new alternative to explain the relationship between chronic stress, the neuroendocrine system, and breast cancer.

## Figures and Tables

**Table 1:** Primers for genes selected to develop cDNA probes.

Gene Name	Primer Sequence ^a^
*CCND1*	F: AATGACCCCGCACGATTTCA R: TGAGGCGGTAGTAGGACAGG
*E-Cadherin*	F: AGTGGGCACAGATGGTGTGA R: TAGGTGGAGTCCCAGGCGTA
*ESR1*	F: TGATTGGTCTCGTCTGGCG R: CATGCCCTCTACACATTTTCCC
*FOS*	F: AAGGAGAATCCGAAGGGAAAGG R: GGCAATCTCGGTCTGCAAAG
*N-Cadherin*	F: TCG ATT GGT TTG ACC ACG G R: GAC GGT TCG CCA TCC AGA C
*PGR*	F: AGGTCTACCCGCCCTATCTC R: AGTAGTTGTGCTGCCCTTCC
*SLUG*	F: GACCCTGGTTGCTTCAAGGA R: TGTTGCAGTGAGGGCAAGAA
*TFF1*	F: TTCTATCCTAATACCATCGACG R: TTTGAGTAGTCAAAGTCAGAGC
*TWIST*	F: TCCGCGTCCCACTAGCA R: AGTTATCCAGCTCCAGAGTCTCTAGAC
*VIMENTIN*	F: TGTCCAAATCGATGTGGATGTTTC R: TTGTACCATTCTTCTGCCTCCTG
*ß-actin*	F: TGCCGACAGGATGCAGAAG R: GCCGATCCACACGGAGTACT

^a^ PCR primer sequence used to generate a product of the indicated size, listed in 5′→3′ orientation. F, forward; R, reverse.

## Data Availability

The data generated in the present study may be requested from the corresponding author. STRING data and download files are freely available under a ‘Creative Commons BY 4.0’ license (https://string-db.org), accessed on 22 March 2023.

## Supplementary Materials

The following supporting information can be downloaded at: https://www.mdpi.com/article/10.3390/biology12111418/s1. Figure S1. Optical density charts for Figure 2; Figure S2. Optical density charts for Figure 5.

## References

[1] Siegel R.L., Miller K.D., Fuchs H.E., Jemal A. (2022). Cancer statistics, 2022. CA A Cancer J. Clin..

[2] Sung H., Ferlay J., Siegel R.L., Laversanne M., Soerjomataram I., Jemal A., Bray F. (2021). Global Cancer Statistics 2020: GLOBOCAN Estimates of Incidence and Mortality Worldwide for 36 Cancers in 185 Countries. CA A Cancer J. Clin..

[3] Tang P., Tse G.M. (2016). Immunohistochemical Surrogates for Molecular Classification of Breast Carcinoma: A 2015 Update. Arch. Pathol. Lab. Med..

[4] Johnson K.S., Conant E.F., Soo M.S. (2020). Molecular Subtypes of Breast Cancer: A Review for Breast Radiologists. J. Breast Imaging.

[5] Lumachi F., Santeufemia D.A., Basso S.M. (2015). Current medical treatment of estrogen receptor-positive breast cancer. World J. Biol. Chem..

[6] Waks A.G., Winer E.P. (2019). Breast Cancer Treatment: A Review. JAMA.

[7] Anbalagan M., Rowan B.G. (2015). Estrogen receptor alpha phosphorylation and its functional impact in human breast cancer. Mol. Cell. Endocrinol..

[8] Yasar P., Ayaz G., User S.D., Gupur G., Muyan M. (2017). Molecular mechanism of estrogen-estrogen receptor signaling. Reprod. Med. Biol..

[9] VanHook A.M. (2010). Ligand-Independent ER Activation. Sci. Signal..

[10] Rudel R.A., Fenton S.E., Ackerman J.M., Euling S.Y., Makris S.L. (2011). Environmental exposures and mammary gland development: State of the science, public health implications, and research recommendations. Environ. Health Perspect..

[11] Cabello G., Valenzuela M., Vilaxa A., Duran V., Rudolph I., Hrepic N., Calaf G. (2001). A rat mammary tumor model induced by the organophosphorous pesticides parathion and malathion, possibly through acetylcholinesterase inhibition. Environ. Health Perspect..

[12] Calaf G.M. (2023). Breast carcinogenesis induced by organophosphorous pesticides. Adv. Pharmacol..

[13] Calaf G.M. (2021). Role of organophosphorous pesticides and acetylcholine in breast carcinogenesis. Semin. Cancer Biol..

[14] Calaf G.M., Echiburu-Chau C., Roy D. (2009). Organophosphorous pesticides and estrogen induce transformation of breast cells affecting p53 and c-Ha-ras genes. Int. J. Oncol..

[15] Bayer G., Wense T. (1936). Über den Nachweis von Hormonen in einzelligen Tieren. Pflüger’s Arch. Gesamte Physiol. Menschen Tiere.

[16] Wessler I., Kirkpatrick C.J., Racke K. (1998). Non-neuronal acetylcholine, a locally acting molecule, widely distributed in biological systems: Expression and function in humans. Pharmacol. Ther..

[17] Spindel E.R. (2012). Muscarinic receptor agonists and antagonists: Effects on cancer. Handbook of Experimental Pharmacology.

[18] Pettersson A., Nilsson L., Nylund G., Khorram-Manesh A., Nordgren S., Delbro D.S. (2009). Is acetylcholine an autocrine/paracrine growth factor via the nicotinic alpha7-receptor subtype in the human colon cancer cell line HT-29?. Eur. J. Pharmacol..

[19] Spindel E.R. (2016). Cholinergic Targets in Lung Cancer. Curr. Pharm. Des..

[20] Cheng K., Samimi R., Xie G., Shant J., Drachenberg C., Wade M., Davis R.J., Nomikos G., Raufman J.P. (2008). Acetylcholine release by human colon cancer cells mediates autocrine stimulation of cell proliferation. Am. J. Physiol. Gastrointest. Liver Physiol..

[21] Xu R., Shang C., Zhao J., Han Y., Liu J., Chen K., Shi W. (2015). Activation of M3 muscarinic receptor by acetylcholine promotes non-small cell lung cancer cell proliferation and invasion via EGFR/PI3K/AKT pathway. Tumour Biol. J. Int. Soc. Oncodev. Biol. Med..

[22] Yu H., Xia H., Tang Q., Xu H., Wei G., Chen Y., Dai X., Gong Q., Bi F. (2017). Acetylcholine acts through M3 muscarinic receptor to activate the EGFR signaling and promotes gastric cancer cell proliferation. Sci. Rep..

[23] Eglen R.M. (2006). Muscarinic receptor subtypes in neuronal and non-neuronal cholinergic function. Auton. Autacoid. Pharmacol..

[24] Abrams P., Andersson K.E., Buccafusco J.J., Chapple C., de Groat W.C., Fryer A.D., Kay G., Laties A., Nathanson N.M., Pasricha P.J. (2006). Muscarinic receptors: Their distribution and function in body systems, and the implications for treating overactive bladder. Br. J. Pharmacol..

[25] Santiago L.J., Abrol R. (2019). Understanding G Protein Selectivity of Muscarinic Acetylcholine Receptors Using Computational Methods. Int. J. Mol. Sci..

[26] Mehedinteanu A.M., Mirea C.S., Stovicek P.O., Schenker M., Stancu M.I., Ciurea A.M., Streba L., Istrate-Ofiteru A.M., Sas T.N., Vere C.C. (2021). Expression of M3 muscarinic acetylcholine receptors in gastric cancer. Rom. J. Morphol. Embryol..

[27] Lin G., Sun L., Wang R., Guo Y., Xie C. (2014). Overexpression of muscarinic receptor 3 promotes metastasis and predicts poor prognosis in non-small-cell lung cancer. J. Thorac. Oncol. Off. Publ. Int. Assoc. Study Lung Cancer.

[28] Larabee S.M., Cheng K., Raufman J.P., Hu S. (2022). Muscarinic receptor activation in colon cancer selectively augments pro-proliferative microRNA-21, microRNA-221 and microRNA-222 expression. PLoS ONE.

[29] Lombardi M.G., Negroni M.P., Pelegrina L.T., Castro M.E., Fiszman G.L., Azar M.E., Morgado C.C., Sales M.E. (2013). Autoantibodies against muscarinic receptors in breast cancer: Their role in tumor angiogenesis. PLoS ONE.

[30] Parnell E.A., Calleja-Macias I.E., Kalantari M., Grando S.A., Bernard H.U. (2012). Muscarinic cholinergic signaling in cervical cancer cells affects cell motility via ERK1/2 signaling. Life Sci..

[31] Cheng K., Shang A.C., Drachenberg C.B., Zhan M., Raufman J.P. (2017). Differential expression of M3 muscarinic receptors in progressive colon neoplasia and metastasis. Oncotarget.

[32] Kuol N., Davidson M., Karakkat J., Filippone R.T., Veale M., Luwor R., Fraser S., Apostolopoulos V., Nurgali K. (2022). Blocking Muscarinic Receptor 3 Attenuates Tumor Growth and Decreases Immunosuppressive and Cholinergic Markers in an Orthotopic Mouse Model of Colorectal Cancer. Int. J. Mol. Sci..

[33] Espanol A.J., Salem A., Di Bari M., Cristofaro I., Sanchez Y., Tata A.M., Sales M.E. (2020). The metronomic combination of paclitaxel with cholinergic agonists inhibits triple negative breast tumor progression. Participation of M2 receptor subtype. PLoS ONE.

[34] Jimenez E., Montiel M. (2005). Activation of MAP kinase by muscarinic cholinergic receptors induces cell proliferation and protein synthesis in human breast cancer cells. J. Cell. Physiol..

[35] Fiszman G.L., Middonno M.C., de la Torre E., Farina M., Espanol A.J., Sales M.E. (2007). Activation of muscarinic cholinergic receptors induces MCF-7 cells proliferation and angiogenesis by stimulating nitric oxide synthase activity. Cancer Biol. Ther..

[36] Ahmed E.A., Alkuwayti M.A., Ibrahim H.M. (2022). Atropine Is a Suppressor of Epithelial-Mesenchymal Transition (EMT) That Reduces Stemness in Drug-Resistant Breast Cancer Cells. Int. J. Mol. Sci..

[37] Sales M.E. (2016). Muscarinic Receptors as Targets for Metronomic Therapy in Breast Cancer. Curr. Pharm. Des..

[38] Espanol A.J., Salem A., Rojo D., Sales M.E. (2015). Participation of non-neuronal muscarinic receptors in the effect of carbachol with paclitaxel on human breast adenocarcinoma cells. Roles of nitric oxide synthase and arginase. Int. Immunopharmacol..

[39] Calaf G.M., Crispin L.A., Munoz J.P., Aguayo F., Bleak T.C. (2022). Muscarinic Receptors Associated with Cancer. Cancers.

[40] Cardoso C.C., Ricardo V.P., Frussa-Filho R., Porto C.S., Abdalla F.M. (2010). Effects of 17 beta-estradiol on expression of muscarinic acetylcholine receptor subtypes and estrogen receptor alpha in rat hippocampus. Eur. J. Pharmacol..

[41] Long C.Y., Hsu C.S., Shao P.L., Liu C.M., Su J.H., Tsai E.M. (2009). Effect of ovariectomy on the gene expression of detrusor muscarinic receptors in female rats. Fertil. Steril..

[42] Rodgers S.P., Bohacek J., Daniel J.M. (2010). Transient estradiol exposure during middle age in ovariectomized rats exerts lasting effects on cognitive function and the hippocampus. Endocrinology.

[43] Matucci R., Bianchi B., Mantelli L., Ghelardini C., Vannelli G.B., Maggi M. (1996). Influence of oestrogens on muscarinic receptor density and contractile response in the guinea-pig uterus. J. Reprod. Fertil..

[44] Abdalla F.M., Marostica E., Picarelli Z.P., Abreu L.C., Avellar M.C., Porto C.S. (2004). Effect of estrogen on muscarinic acetylcholine receptor expression in rat myometrium. Mol. Cell. Endocrinol..

[45] Munoz J.P., Araya-Osorio R., Mera-Adasme R., Calaf G.M. (2023). Glyphosate mimics 17beta-estradiol effects promoting estrogen receptor alpha activity in breast cancer cells. Chemosphere.

[46] Livak K.J., Schmittgen T.D. (2001). Analysis of relative gene expression data using real-time quantitative PCR and the 2(-Delta Delta C(T)) Method. Methods.

[47] Calaf G.M., Roy D., Hei T.K. (2005). Immunochemical analysis of protein expression in breast epithelial cells transformed by estrogens and high linear energy transfer (LET) radiation. Histochem. Cell Biol..

[48] Gee K.R., Brown K.A., Chen W.N., Bishop-Stewart J., Gray D., Johnson I. (2000). Chemical and physiological characterization of fluo-4 Ca^2+^-indicator dyes. Cell Calcium.

[49] Volpicelli L.A., Levey A.I. (2004). Muscarinic acetylcholine receptor subtypes in cerebral cortex and hippocampus. Prog. Brain Res..

[50] Gomez A., Martos F., Bellido I., Marquez E., Garcia A.J., Pavia J., Sanchez de la Cuesta F. (1992). Muscarinic receptor subtypes in human and rat colon smooth muscle. Biochem. Pharmacol..

[51] Uchiyama T., Chess-Williams R. (2004). Muscarinic receptor subtypes of the bladder and gastrointestinal tract. J. Smooth Muscle Res..

[52] Hegde S.S., Eglen R.M. (1999). Muscarinic receptor subtypes modulating smooth muscle contractility in the urinary bladder. Life Sci..

[53] Wang Z., Shi H., Wang H. (2004). Functional M3 muscarinic acetylcholine receptors in mammalian hearts. Br. J. Pharmacol..

[54] Minette P.A., Barnes P.J. (1990). Muscarinic receptor subtypes in lung. Clinical implications. Am. Rev. Respir. Dis..

[55] Nietgen G.W., Schmidt J., Hesse L., Honemann C.W., Durieux M.E. (1999). Muscarinic receptor functioning and distribution in the eye: Molecular basis and implications for clinical diagnosis and therapy. Eye.

[56] Walch L., Brink C., Norel X. (2001). The muscarinic receptor subtypes in human blood vessels. Therapie.

[57] Español A.J., Sales M.E. (2004). Different muscarinc receptors are involved in the proliferation of murine mammary adenocarcinoma cell lines. Int. J. Mol. Med..

[58] Hösli E., Hösli L. (1999). Cellular localization of estrogen receptors on neurones in various regions of cultured rat CNS: Coexistence with cholinergic and galanin receptors. Int. J. Dev. Neurosci..

[59] Cardoso C.C., Pereira R.T., Koyama C.A., Porto C.S., Abdalla F.M. (2004). Effects of estrogen on muscarinic acetylcholine receptors in the rat hippocampus. Neuroendocrinology.

[60] Liu H.M., Ma L.L., Li C., Cao B., Jiang Y., Han L., Xu R., Lin J., Zhang D. (2022). The molecular mechanism of chronic stress affecting the occurrence and development of breast cancer and potential drug therapy. Transl. Oncol..

[61] Bennesch M.A., Picard D. (2015). Minireview: Tipping the balance: Ligand-independent activation of steroid receptors. Mol. Endocrinol..

[62] Fuentes N., Silveyra P. (2019). Estrogen receptor signaling mechanisms. Adv. Protein Chem. Struct. Biol..

