# Supplementary figures and images for: Acetylcholine, Another Factor in Breast Cancer
Source: Biology (Basel). 2023 Nov 11;12(11):1418. doi: 10.3390/biology12111418 (PMC10669196; doi:10.3390/biology12111418)

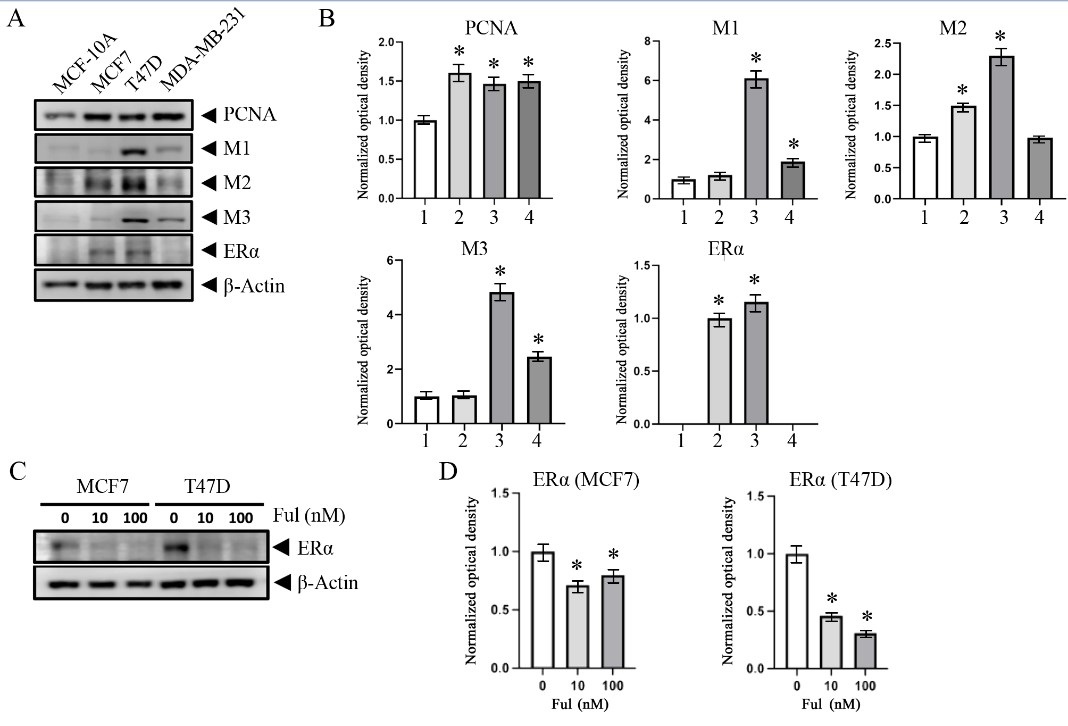

Supplement: Supplementary file 1 [file biology-12-01418-s001.zip › Figure S2.jpg]

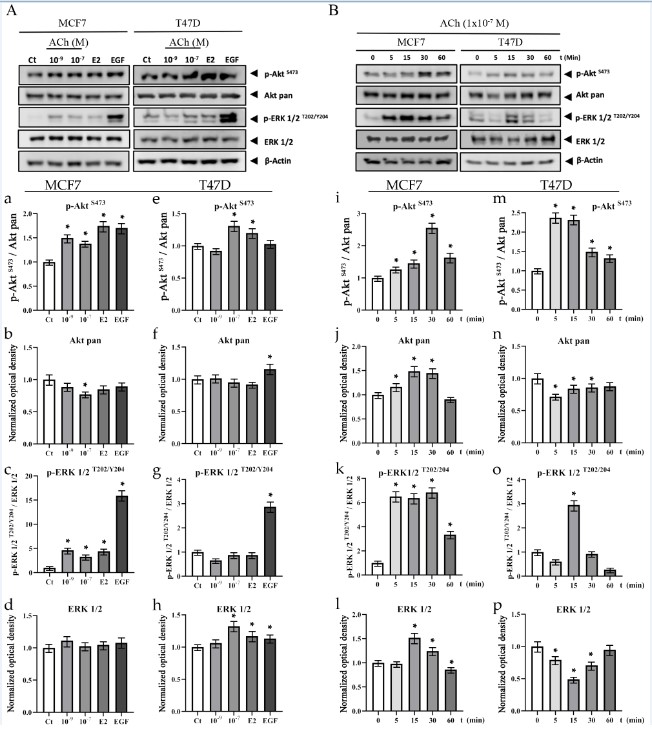

Supplement: Supplementary file 1 [file biology-12-01418-s001.zip › Figure S5.jpg]
